# Experimental evaluation of the healing potential of *Sesuvium portulacastrum* in excisional wounds in wistar rats

**DOI:** 10.3389/fbioe.2025.1707625

**Published:** 2026-02-17

**Authors:** Kelly Kercy Nogueira Da Silva, Gislainy Luciana Gomes Câmara, Salvador Viana Gomes Júnior, Camila Gomes Fernandes De Souza, Kizzy Millenn De Freitas, Amália Cinthia Meneses Do Rego, Irami Araújo Filho, Roque Ribeiro da Silva Júnior, Thales Allyrio Araújo De Medeiros Fernandes, José Rodolfo Lopes de Paiva Cavalcanti, Fausto Pierdoná Guzen

**Affiliations:** 1 Postgraduate Program in Biotechnology, Health School, Potiguar University (UnP), Natal, Brazil; 2 Laboratory Experimental Neurology, Department of Biomedical Sciences, Faculty of Health Sciences, State University of Rio Grande do Norte (UERN), Mossoró, Brazil; 3 Postgraduate Program in Physiological Sciences, Department of Biomedical Sciences, Faculty of Health Sciences, State University Rio Grande do Norte (UERN), Mossoró, Brazil; 4 Postgraduate Program in Health and Society, Department of Biomedical Sciences, Faculty of Health Sciences, University of the State of Rio Grande do Norte (UERN), Mossoró, Brazil

**Keywords:** *Sesuvium portulacastrum*, plant, wound, treatment, extracts, healing, skin

## Abstract

**Background:**

*Sesuvium portulacastrum* (“Pirrixiu”) is a halophytic plant adapted to saline environments with potential wound-healing properties.

**Objective:**

To evaluate the wound-healing efficacy of a 10% macerated *S. portulacastrum* gel compared with the topical antibiotic Nebacetin in Wistar rats with standardized excisional wounds.

**Methods:**

Experimental, completely randomized study in Wistar rats. Wound area reduction was measured daily. The association between time and wound closure was assessed by linear regression. Histological evaluation (hematoxylin and eosin; Masson’s trichrome) examined inflammation, collagen deposition, angiogenesis/vascularization, and re-epithelialization.

**Results:**

Time was strongly associated with wound closure (correlation coefficient > 0.80; p < 0.05). *S. portulacastrum*–treated groups achieved mean wound area reductions of up to 75% during the experimental period and demonstrated significantly greater collagen deposition and re-epithelialization, comparable to the Nebacetin-treated group (p < 0.05). Angiogenesis/vascularization did not differ significantly between groups (p > 0.05). Inflammation was significantly reduced compared with the positive control (p < 0.05). No adverse events or signs of infection or stress were observed.

**Conclusion:**

A 10% S. portulacastrum gel promoted wound healing with enhanced collagen deposition and re-epithelialization, showing effects comparable to Nebacetin. The findings support S. portulacastrum as a promising, low-cost, and potentially sustainable therapeutic alternative and reinforce the value of Caatinga biodiversity.

## 1 Introduction

The use of medicinal plants is among humanity’s oldest therapeutic practices, with records dating back to prehistory, when early communities relied on plant species based on empirical knowledge. Currently, it is estimated that more than 350,000 plant species have been catalogued, many of which exhibit substantial therapeutic and pharmacological potential (Nascimento-Júnior et al., 2021). Scientific interest in these plants has evolved in parallel with advances in chemistry and pharmacology, with particular emphasis on secondary metabolites—such as essential oils, flavonoids, and terpenes—which are widely used in the production of herbal medicines and in traditional medical practices (Wu et al., 2025).

Within the context of Brazilian biodiversity, the Caatinga biome stands out as an exclusively national domain that harbors species adapted to the extreme conditions of the northeastern semiarid region, including high temperatures and prolonged droughts (Barbosa and Gomes, 2022). Despite these adversities, the Caatinga sustains a rich diversity of endemic species, many of which are traditionally used by local populations for disease treatment. Species with distinct morphological adaptations—such as deep root systems and succulent stems—are particularly notable for their resilience to arid conditions (Roque et al., 2010; Albuquerque, 2010; Cordeiro and Félix, 2014).

Among these species, halophytes—plants that thrive in high-salinity environments—have attracted considerable interest in agriculture, ecology, and biotechnology. These plants exhibit complex salt-tolerance mechanisms, including the synthesis of osmoprotectants such as proline, betaine, and sorbitol; salt secretion via specialized glands; and anatomical adaptations that reduce water loss and optimize water uptake (Grigore and Vicente, 2023; Rahman et al., 2021; Meng et al., 2018).

Research on halophytes has become even more relevant in light of the increasing salinization of soils driven by inadequate agricultural practices and climate change. Such species are regarded as promising solutions for cultivation in marginal soils and for the restoration of degraded areas, and they provide valuable genetic resources for the development of more resilient crop varieties (Rahman et al., 2021). Garcia-Caparros et al. (2023) emphasize the high economic and environmental potential of these plants, with applications ranging from phytoremediation to the production of food, medicines, animal feed, and biofuels—versatility that aligns with current demands for sustainable solutions.

In this context, *S. portulacastrum* (Aizoaceae) merits particular attention. The essential oil extracted from its leaves contains, among other constituents, α-pinene, limonene, 1,8-cineole, trans-caryophyllene, and α-humulene (Magwa et al., 2006). In addition, phytochemical reports describe the presence of flavonoids, tannins, saponins, and a relatively high abundance of terpenoids in non-volatile extracts, collectively suggesting pharmacologically relevant activities, particularly antioxidant and antimicrobial effects (Purushothaman et al., 2024).

At the bioactive level, leaf extracts exhibit *in vitro* antibacterial and antioxidant activity, the magnitude of which depends on the solvent and fraction analyzed (Mehmood et al., 2022). Additional studies have also demonstrated antimicrobial effects against diverse panels of microorganisms (Valdivieso-Ugarte et al., 2019). Taken together, the terpenoid composition of the essential oil and the presence of phenolic compounds provide a plausible mechanistic basis for antioxidant, anti-inflammatory, and antimicrobial effects. Nevertheless, chromatographic profiling (e.g., LC–MS/MS) and bioassay-guided fractionation remain necessary to link specific compounds to relevant signaling pathways, thereby strengthening translation to wound healing and other indications (Magwa et al., 2006; Purushothaman et al., 2024; Mehmood et al., 2022; Valdivieso-Ugarte et al., 2019 ).

However, the commercialization of medicinal plant–based products still occurs largely in informal settings, such as open-air markets, often without regulation or quality control, which poses risks to public health. Rodrigues et al. (2017) warn against the indiscriminate use of these products and underscore the need for scientific research to ensure their safety and efficacy. Despite the therapeutic potential of the Caatinga flora, systematic studies of its species remain incipient in Brazil, highlighting the need for further investigation. In this scenario, the present study aims to experimentally evaluate the wound-healing potential of the halophyte *S. portulacastrum* in induced excisional wounds in Wistar rats, thereby contributing to scientific knowledge on the safe and effective use of plants adapted to saline environments.

## 2 Materials and methods

### 2.1 Study type and experimental design

**FIGURE 1:**
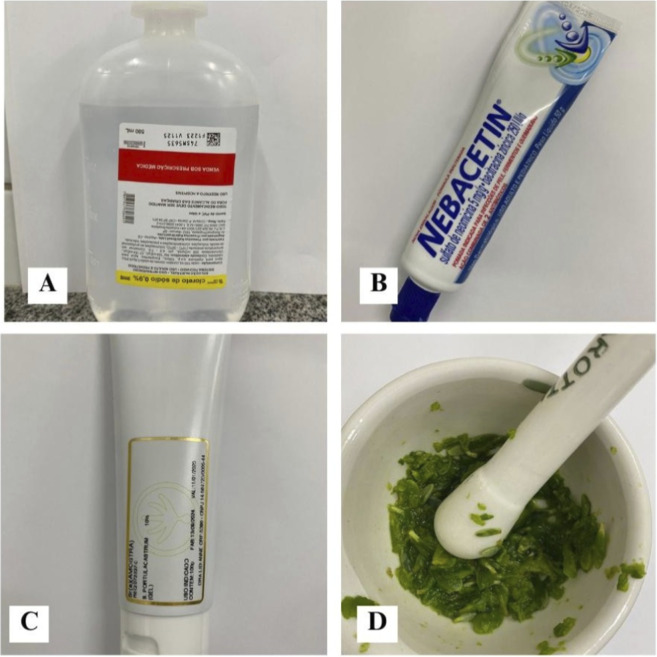
Representation of the treatments used in the experiment. Legend: **(A)** Saline solution (negative control); **(B)** Nebacetin® (positive control); **(C)** Gel formulated with 10% *Sesuvium portulacastrum* extract; **(D)** Macerated *Sesuvium portulacastrum* used as an alternative phytotherapeutic intervention.

This study was designed as an experimental investigation with a quantitative approach. Its primary objective was to evaluate the wound-healing potential of *Sesuvium portulacastrum* in excisional wounds induced in Wistar rats and to compare its effects with those of conventional treatments. As shown in Figure 1, the adopted experimental design enabled appropriate allocation of treatments across animals, minimized bias, and ensured the internal validity of the findings. Quantitative analyses of the dependent variables, such as wound dimensions, were conducted using linear regression models, alongside correlation coefficients to assess the association between time and healing (Montgomery et al., 2021). The project complied with all applicable regulations for animal research, and all procedures were reviewed and approved by the Ethics Committee on Animal Use of the State University of Rio Grande do Norte (UERN), under decision CEEA/UERN No. 004/2024.

### 2.2 Experimental animals

Thirty-two male Wistar rats (*Rattus norvegicus*), aged 50 days, with a mean body weight of 290 ± 30 g, were used. Before the experimental procedures, the animals were housed in the institutional animal facility in collective plastic cages (30 × 16 × 19 cm), with a maximum of three animals per cage, under controlled conditions (22 °C ± 2 °C). Standard chow and tap water were provided *ad libitum*. Animals were allocated to experimental groups of six individuals each, based on predefined inclusion criteria (apparently good health and body weight within the specified range) and exclusion criteria (disease, abnormal behavior, or body weight outside the established parameters). Each animal was individually marked by a trained technician who did not participate in the evaluations, thereby supporting blinding of outcome assessment.

### 2.3 Environmental conditions and husbandry

The animals were maintained under controlled environmental conditions (22 °C–24 °C) in standard polypropylene cages containing wood shavings as bedding. A 12 h light/dark cycle was used. Animals received Supralab® pelleted rodent chow and filtered, purified drinking water *ad libitum*. Drinking water was supplied in bottle-type dispensers and replaced daily. Environmental parameters were routinely monitored to ensure stability of the experimental setting and to minimize external sources of variability that could influence the outcomes.

The commercial chow had the following composition: ground corn*, wheat bran, rice bran, soybean meal**, ground alfalfa hay, limestone, sodium chloride, DL-methionine, bentonite, vitamin A, vitamin C, vitamin D_3_, vitamin E, vitamin K_3_, vitamin B_1_, vitamin B_2_, niacin (nicotinic acid), D-calcium pantothenate, vitamin B_6_, biotin, folic acid, vitamin B_12_, choline chloride, copper sulfate, iron sulfate, calcium iodate, manganese sulfate, sodium selenite, zinc oxide, ethoxyquin, calcium propionate, potassium sorbate, and kaolin.

Donor species for the genetic material declared in the ingredients were as follows:

- *Agrobacterium tumefaciens*, *Bacillus subtilis*, *Bacillus thuringiensis*, *Diabrotica virgifera*, *Dicossoma* sp., *Escherichia coli*, *Sphingobium herbicidovorans*, *Stenotrophomonas maltophilia*, *Streptomyces viridochromogenes*, *Thermococcales* spp., and *Zea mays.*
- *Agrobacterium tumefaciens*, *Arabidopsis thaliana*, *Bacillus thuringiensis*, *Delftia acidovorans*, *Glycine max*, *Helianthus annuus*, *Pseudomonas fluorescens*, *Stenotrophomonas maltophilia*, *Streptomyces hygroscopicus*, *Streptomyces viridochromogenes*, and *Zea mays.*

Technical specifications (minimum or maximum values per kg of feed): moisture (max.) 100 g/kg (10%); crude protein (min.) 220 g/kg (22%); ether extract (min.) 40 g/kg (4%); crude fiber (max.) 70 g/kg (7%); ash (max.) 100 g/kg (10%); calcium (min.) 8,000 mg/kg (0.8%) and calcium (max.) 12 g/kg (1.2%); phosphorus (min.) 4,000 mg/kg (0.4%); methionine (min.) 4,200 mg/kg; lysine (min.) 10 g/kg; vitamin A (min.) 12,800 IU/kg; vitamin C (min.) 150 mg/kg; vitamin D_3_ (min.) 2,800 IU/kg; vitamin E (min.) 48 IU/kg; vitamin K_3_ (min.) 4.8 mg/kg; vitamin B_1_ (min.) 2 mg/kg; vitamin B_2_ (min.) 6.4 mg/kg; vitamin B_3_ (niacin, min.) 56 mg/kg; vitamin B_5_ (pantothenic acid, min.) 12 mg/kg; vitamin B_6_ (min.) 3.2 mg/kg; vitamin B7 (biotin, min.) 0.2 mg/kg; vitamin B_9_ (folic acid, min.) 2 mg/kg; vitamin B_12_ (min.) 32 μg/kg; choline (min.) 1,500 mg/kg; copper (min.) 9.6 mg/kg; iron (min.) 35 mg/kg; iodine (min.) 1.2 mg/kg; manganese (min) 96 mg/kg; selenium (min.) 0.36 mg/kg; and zinc (min.) 84 mg/kg.

### 2.4 Phytochemical profile

*Sesuvium portulacastrum* contains phenols, saponins, steroids, tannins, and terpenoids, which have been associated with antioxidant activity, including a dose-dependent effect on nitric oxide radical scavenging (Mukundh et al., 2024). In addition, alkaloids, saponins, tannins (5% ferric chloride), terpenoids (2,4-dinitrophenylhydrazine), and steroids have been identified in its phytochemical profile (Al-Azzawi et al., 2012).

### 2.5 Wound induction and treatments

Animals were anesthetized intraperitoneally with ketamine hydrochloride (90 mg/kg) and xylazine (15 mg/kg). After a surgical plane of anesthesia was achieved, the dorsal region was shaved and aseptically prepared. Standardized circular full-thickness excisional wounds (1 cm in diameter) were created using a scalpel. Immediately after wound creation, the assigned treatment was applied according to group allocation, once daily for 14 days.

### 2.6 Formulation and allocation of experimental groups

*Sesuvium portulacastrum* was collected fresh (*in natura*) in the municipality of Grossos, Rio Grande do Norte, Brazil. Species identification was confirmed, and the specimen was catalogued by the Herbology Department of the Federal Rural University of the Semi-Arid (UFERSA).

After washing under running water with neutral soap, the plant material was dried in a ventilated oven at 68 °C for 24 h and then ground using a mortar and pestle to obtain a macerate. For the relevant treatment group, the macerate was applied to the wounds every 12 h until euthanasia. For gel preparation, the macerated material was incorporated into a formulation containing 1% Carbopol 940, with the addition of propylene glycol (5%) and a paraben preservative; the pH was adjusted to 6.0 using sodium hydroxide. The gel was homogenized under constant stirring to ensure formulation uniformity, yielding a final concentration of 10% plant material.

Animals were assigned to the following groups: (A) 0.9% saline solution (negative control); (B) Nebacetin® (conventional topical antibiotic; positive control); (C) *S. portulacastrum* gel; and (D) pure macerated *S. portulacastrum*, applied directly to the wound every 12 h until euthanasia. Animals were randomized to groups by simple random draw to ensure homogeneous distribution. Wound assessments were performed by blinded evaluators who were unaware of group allocation. To maintain blinding, animals were marked by a technician who did not participate in the evaluations.

### 2.7 Post-experimental procedures and tissue processing

At the end of the protocol, animals were anesthetized prior to euthanasia by intraperitoneal injection of ketamine (50 mg/kg) and xylazine (10 mg/kg). Euthanasia was then performed by administering higher intraperitoneal doses of ketamine (150 mg/kg) and xylazine (30 mg/kg). Subsequently, skin samples from the wound region were excised and processed for sectioning. Coronal sections (30 μm) were obtained in a cryostat using Tissue-Tek embedding medium. Sections were stored in 0.1 M phosphate buffer (pH 7.4) and kept at −20 °C until histochemical analysis by light microscopy, using hematoxylin and eosin (H&E) and Masson’s trichrome (MT) staining.

### 2.8 Statistical analysis

Quantitative data on wound area and perimeter, fibroblast counts, collagen fiber deposition, and the presence of abscesses were tabulated and analyzed using SPSS (version 20.0). Linear regression was used to model the healing trajectory. Fisher’s exact test with Bonferroni correction was applied for between-group comparisons of wound area, inflammation, crust formation, collagen deposition, vascularization, and re-epithelialization. Pearson’s correlation coefficient was used to assess the association between time and the reduction in wound area. Statistical significance was set at *p* < 0.05.

### 2.9 Ethical considerations

The study was approved by the Animal Experimentation Ethics Committee (CEEA) under protocol No. 004/2024 and was conducted in accordance with national and international guidelines for the use of animals in research.

## 3 Results

**FIGURE 2:**
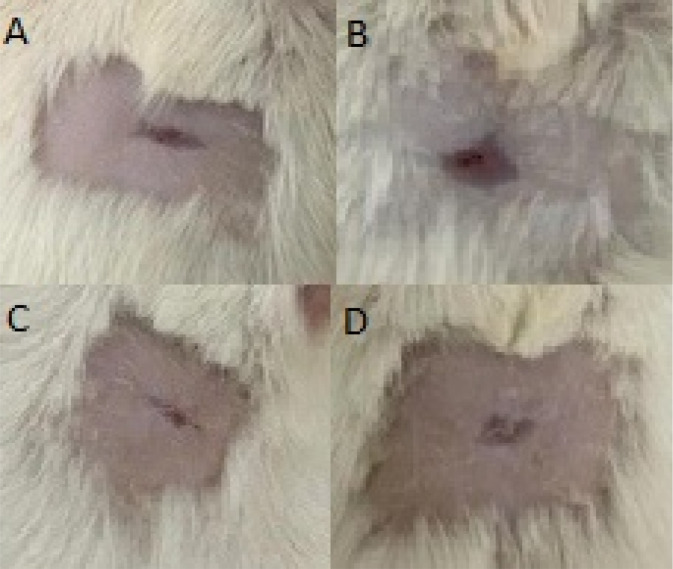
Photographs of Wistar rats at the end of treatment, showing wound appearance in the different groups. Legend: **(A)** Saline Solution group, with a larger lesion and irregular edges, indicating incomplete healing. **(B)** Positive Control group (Nebacetin®), with advanced healing and near-complete wound closure. **(C)** Group treated with *S. portulacastrum* gel, showing a marked reduction in lesion area and partial reepithelialization. **(D)** Group treated with *S. portulacastrum* macerate, with complete wound closure and uniformly colored skin, suggesting effective healing.

The analysis of the area variable across five measurements (M1–M5) was conducted to characterize the trajectory of wound healing in the positive control, saline solution, SP gel, and SP macerate groups. Although visual differences were apparent in the graphs, no statistically significant between-group differences were detected at any time point (*p* > 0.05). At M1, mean values ranged from 107.9 (SP Gel) to 119.4 (saline solution), without meaningful separation between groups. At M2, wound area decreased in the positive control (PC) and SP gel (G-SP) groups (both 74.9), whereas the saline solution (SS) group remained higher (107.9). At M3, SP Gel (23.6) and SP Macerate (31.6) showed the lowest means compared with Saline Solution (43.1); however, these differences were not statistically significant. At M4, the saline solution group exhibited the highest mean area (17.4), while the remaining groups ranged from 7.5 to 9.4. At M5, SP Gel (2.8) and SP Macerate (2.7) again presented the lowest values, yet without statistically significant differences among groups. As shown in Figure 2, despite trends suggesting lower wound area in the SP Gel and SP Macerate groups, the results do not support a statistically demonstrable advantage of these treatments. Overall, all groups displayed comparable reductions in wound area over time, indicating similar effects across interventions under the conditions evaluated.

### 3.1 Area

Statistical analysis of the wound area variable using Fisher’s test did not identify statistically significant differences between the Positive Control group and any other experimental group. Relative to the Saline Solution group, the mean difference was 1.01667 (standard error = 1.33117; *p* = 0.451); relative to SP Gel, −0.05000 (*p* = 0.970); and relative to SP Macerate, 2.15667 (*p* = 0.134); all comparisons were non-significant (*p* > 0.05).

When the Saline Solution group was compared with the remaining groups, the mean difference was 0.96667 versus SP Gel (*p* = 0.474) and 3.17333 versus SP Macerate (standard error = 1.39615; *p* = 0.031), with the latter representing the only nominally significant difference (*p* < 0.05). Thus, SP Macerate differed significantly only in comparison with Saline Solution prior to adjustment. SP Gel also showed no significant differences when compared with the Positive Control (*p* = 0.970), Saline Solution (*p* = 0.474), or SP Macerate (*p* = 0.125).

**FIGURE 3:**
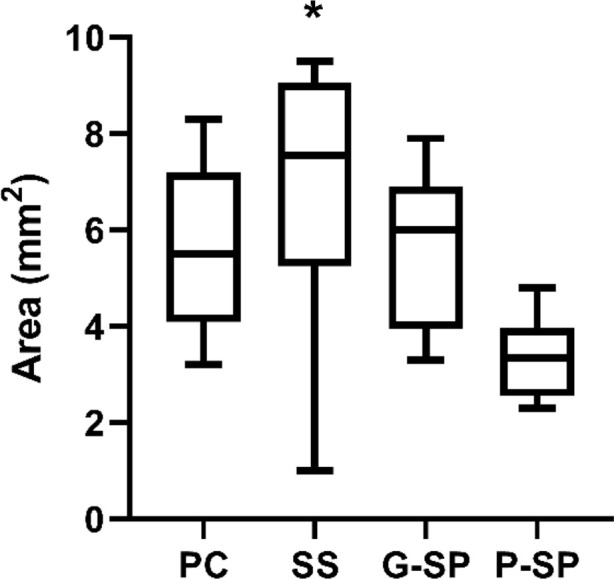
Comparative analysis of the wound area variable among groups. Legend: Comparison of area (mm^2^) among the experimental groups. PC, Positive Control; SS, Saline Solution; G-SP, SP Gel; P-SP, SP Paste. The asterisk (*) denotes a statistically significant difference (p < 0.05). Notably, a statistically significant difference was observed between SS and P-SP.

For comparisons involving SP Macerate, only the contrast with Saline Solution reached nominal significance; comparisons with the Positive Control (*p* = 0.134) and SP Gel (*p* = 0.125) were not significant (*p* > 0.05). However, after Bonferroni correction for multiple comparisons, all adjusted *p*-values exceeded 0.05, indicating that the initially observed difference no longer met the threshold for statistical significance. As shown in Figure 3, overall, no statistically significant differences were detected among groups with respect to wound area after correction for multiple testing.

### 3.2 Inflammation

Statistical analysis of the inflammation variable using Fisher’s test identified a statistically significant difference only between the Positive Control group and the Saline Solution group (mean difference = 1.6667; standard error = 0.3490; *p* = 0.000). This finding was confirmed after Bonferroni adjustment (*p* = 0.01). Comparisons with the SP Gel group (*p* = 0.066) and the SP Macerate group (*p* = 0.529) were not statistically significant.

When the Saline Solution group was compared with the other groups, Fisher’s test indicated significant differences versus the Positive Control (*p* = 0.000), SP Gel (mean difference = −1.0000; *p* = 0.008), and SP Macerate (mean difference = 1.4333; *p* = 0.001). However, after Bonferroni correction, only the comparisons with the Positive Control (*p* = 0.001) and SP Macerate (*p* = 0.008) remained statistically significant; the comparison with SP Gel was no longer significant (*p* = 0.117).

For SP Gel, the only nominally significant result under Fisher’s test was observed in comparison with Saline Solution (*p* = 0.008); however, this difference did not remain statistically significant after Bonferroni adjustment. The remaining comparisons, with the Positive Control (*p* = 0.66) and SP Macerate (*p* = 0.246), were not significant.

**FIGURE 4:**
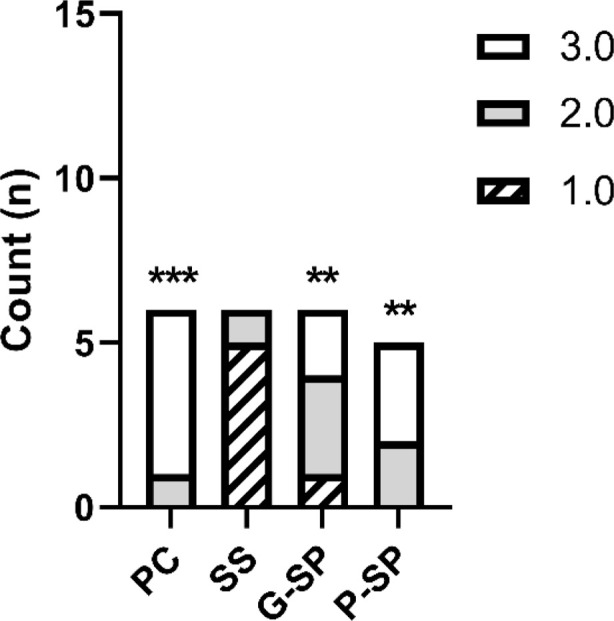
The graph represents the mean scores for the inflammation variable across the different experimental groups. Legend: Distribution of inflammation scores among the experimental groups. Inflammatory intensity was assessed using an ordinal scale, in which Score 1 corresponds to severe inflammation, Score 2 to moderate inflammation, and Score 3 to mild inflammation. Statistical analysis was performed using Fisher’s exact test with Bonferroni correction (α = 0.05). Statistically significant differences were observed between the Positive Control (PC) and Saline Solution (SS) (*p* < 0.001), between SS and SP Gel (G-SP) (*p* = 0.008), and between SS and SP Paste (P-SP) (*p* = 0.001). Overall, the results suggest a better anti-inflammatory response in the SP Gel and SP Paste treatments compared with the Saline Solution group. In the graph, asterisks indicate statistically significant comparisons: *** for *p* < 0.001 and ** for *p* = 0.001.

For SP Macerate, Fisher’s test indicated a significant difference only when compared with Saline Solution (*p* = 0.001), and this result remained significant after Bonferroni correction (*p* = 0.008). Comparisons with the Positive Control and SP Gel were not statistically significant. As shown in Figure 4, the only consistent statistically significant contrast was between SP Macerate and Saline Solution, and this pattern is illustrated in the corresponding graph.

Inflammation is characterized by an increase in inflammatory cells in the skin as part of the immune response to injury, with migration of these cells to the affected site to eliminate pathogens and clear damaged tissue.

### 3.3 Scab formation

Analysis of the scab variable using Fisher’s test did not identify statistically significant differences between the Positive Control group and any other experimental group. Comparisons with the Saline Solution and SP Gel groups both yielded *p* = 0.430, whereas the comparison with the SP Macerate group yielded *p* = 0.368; all results were non-significant (*p* > 0.05). These findings indicate no detectable differences in scab formation between the Positive Control group and the remaining groups.

Similarly, no statistically significant differences were observed when the Saline Solution group was compared with the other groups (*p* = 0.430 vs. Positive Control; *p* = 1.000 vs. SP Gel; *p* = 0.880 vs. SP Macerate). The SP Gel group also showed no significant differences when compared with Positive Control (*p* = 0.430), Saline Solution (*p* = 1.000), or SP Macerate (*p* = 0.880). Using SP Macerate as the reference group likewise yielded no significant contrasts (*p* = 0.368 vs. Positive Control; *p* = 0.880 vs. Saline Solution; *p* = 0.880 vs. SP Gel).

**FIGURE 5:**
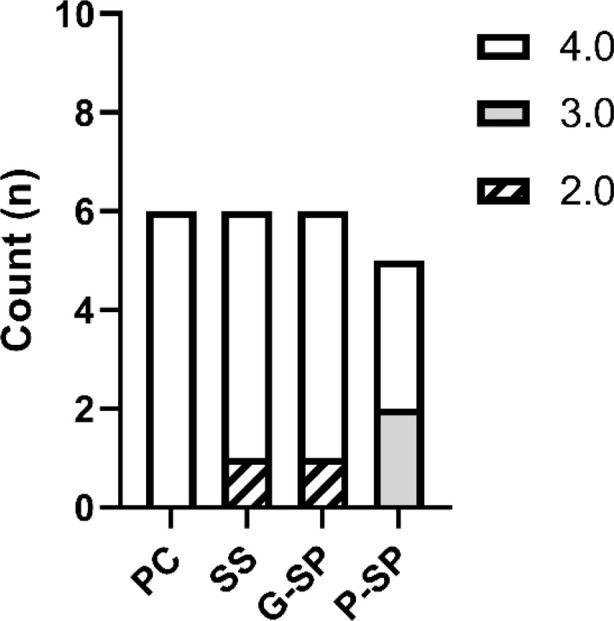
The graph shows the distribution of the scab formation variable among the Positive Control, Saline Solution, SP Gel, and SP Macerate groups, according to an ordinal intensity scale. Legend: Positive Control (PC), Saline Solution (SS), SP Gel (G-SP), SP Paste (P-SP). Frequency of crust formation across experimental groups. Scab formation was evaluated using an ordinal scale from 2 to 4. Score 4 (absence or minimal crust formation) was the most prevalent across all groups, suggesting a favorable healing response. Score 2 (moderate formation) showed the lowest frequency, while Score 3 (mild formation) had intermediate occurrence. Statistical analysis revealed no significant differences between the treated and control groups (p-value >0.05), indicating that the predominance of minimal crust formation was consistent throughout the experiment.

After Bonferroni correction for multiple comparisons, all adjusted *p*-values were 1.000, further reinforcing the absence of statistically significant differences in scab formation across groups. As shown in Figure 5, these results indicate broadly similar scab outcomes among treatments.

### 3.4 Collagen

In the analysis of the collagen variable, using the Positive Control group as the reference, Fisher’s test did not detect statistically significant differences in any comparison. The contrast with the Saline Solution group yielded *p* = 0.085, which approached the significance threshold but remained above the predefined cutoff (*p* ≤ 0.05). Comparisons with the SP Gel (*p* = 0.379) and SP Macerate (*p* = 0.800) groups were also non-significant.

Using the Saline Solution group as the reference produced a similar pattern. The comparison with the Positive Control group again yielded *p* = 0.085. The contrast with SP Gel was *p* = 0.379, indicating no evidence of a difference. The comparison with SP Macerate yielded *p* = 0.060, which was slightly lower but still above the significance threshold, suggesting at most a non-significant trend.

**FIGURE 6:**
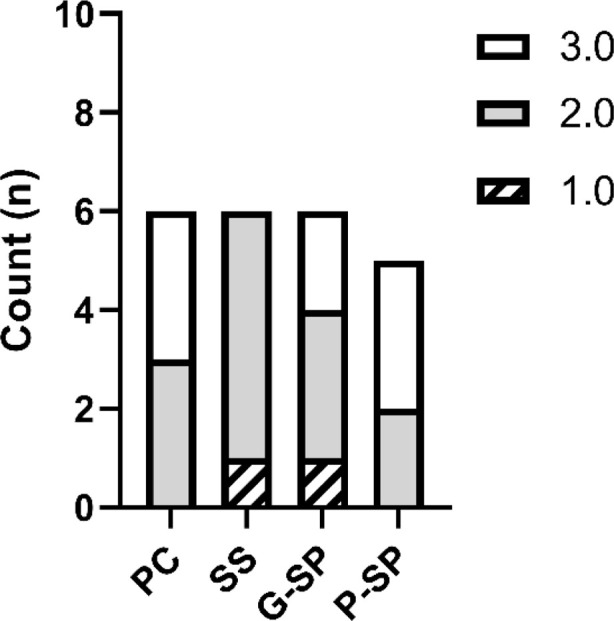
The graph presents the analysis of the collagen variable in the Positive Control, Saline Solution, SP Gel, and SP Macerate groups. Legend: Distribution of collagen deposition levels across experimental groups. Collagen density was evaluated using an ordinal scale: Score 1 indicates high deposition, Score 2 indicates moderate deposition, and Score 3 represents low deposition. A predominance of Score 2 was observed in the Positive Control, Saline Solution, and Gel SP groups, reflecting moderate collagen levels in these treatments. In contrast, the Pasta SP group (Macerate) showed a higher frequency of Score 3, suggesting lower collagen deposition. Despite these observed variations, statistical analysis revealed no significant differences between the groups (p-value >0.05).

When SP Gel was used as the reference, all comparisons remained non-significant: Positive Control and Saline Solution (*p* = 0.379 for both) and SP Macerate (*p* = 0.278). Likewise, when SP Macerate was used as the reference, Fisher’s test showed no statistically significant differences versus Positive Control (*p* = 0.800), Saline Solution (*p* = 0.060), or SP Gel (*p* = 0.278). Thus, even comparisons with *p*-values near 0.05 (e.g., 0.060) did not reach statistical significance. As shown in Figure 6, overall, collagen deposition did not differ significantly among treatments.

After Bonferroni correction for multiple comparisons, none of the pairwise contrasts met the criterion for statistical significance, corroborating the unadjusted results and indicating no statistically significant differences in collagen deposition across groups.

### 3.5 Vascularization

In the analysis of the vascularization variable, using the Positive Control group as the reference, Fisher’s test indicated no statistically significant differences in any comparison. The contrast with the Saline Solution group yielded *p* = 0.088, which was slightly above the significance threshold (*p* ≤ 0.05) and therefore suggests, at most, a non-significant trend. Comparisons with SP Gel (*p* = 0.662) and SP Macerate (*p* = 0.362) were also non-significant.

Using the Saline Solution group as the reference, results likewise showed no statistically significant differences: *p* = 0.088 versus Positive Control, *p* = 0.195 versus SP Gel, and *p* = 0.454 versus SP Macerate, all exceeding the significance cutoff. When SP Gel was used as the reference, no significant contrasts were observed versus Positive Control (*p* = 0.662), Saline Solution (*p* = 0.195), or SP Macerate (*p* = 0.617). Similarly, when SP Macerate was used as the reference, all pairwise comparisons remained non-significant: Positive Control (*p* = 0.362), Saline Solution (*p* = 0.454), and SP Gel (*p* = 0.617).

**FIGURE 7:**
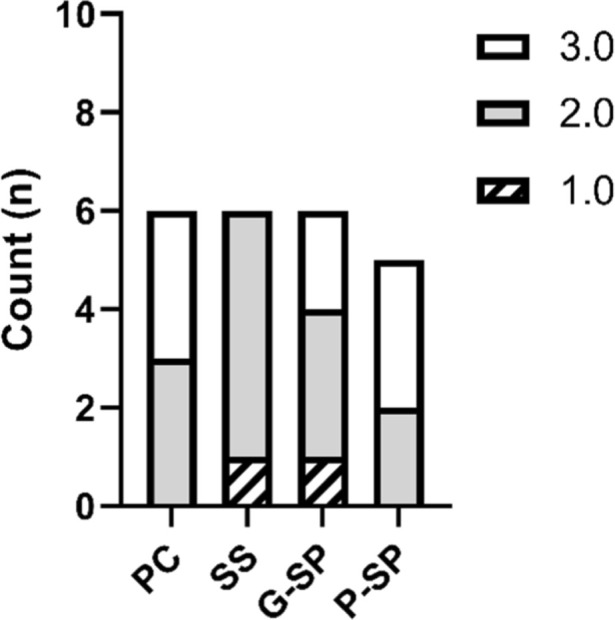
The graph presents the analysis of tissue vascularization in the Positive Control, Saline Solution, SP Gel, and SP Macerate groups. Legend: Tissue vascularization levels across experimental groups. Vascular density was assessed using an ordinal scale: Score 1 represents marked vascularization, Score 2 indicates moderate vascularization, and Score 3 corresponds to mild vascularization. A predominance of Score 2 was observed in the Positive Control and Pasta SP (Macerate) groups, suggesting moderate vascularization. The Saline Solution group showed a higher frequency of Score 1, while the Gel SP group demonstrated a proportional distribution across all scores. Despite these observed variations, statistical analysis revealed no significant differences among the groups (p-value >0.05).

After Bonferroni correction for multiple comparisons, all adjusted *p*-values were 1.000, confirming the absence of statistically significant differences. As shown in Figure 7, vascularization did not differ significantly among the analyzed groups under the conditions evaluated.

### 3.6 Epithelialization

In the analysis of the epithelialization variable, using the Positive Control group as the reference, Fisher’s test identified a statistically significant difference only in comparison with the Saline Solution group (*p* = 0.017). Comparisons with SP Gel (*p* = 0.531) and SP Macerate (*p* = 1.000) were not statistically significant.

**FIGURE 8:**
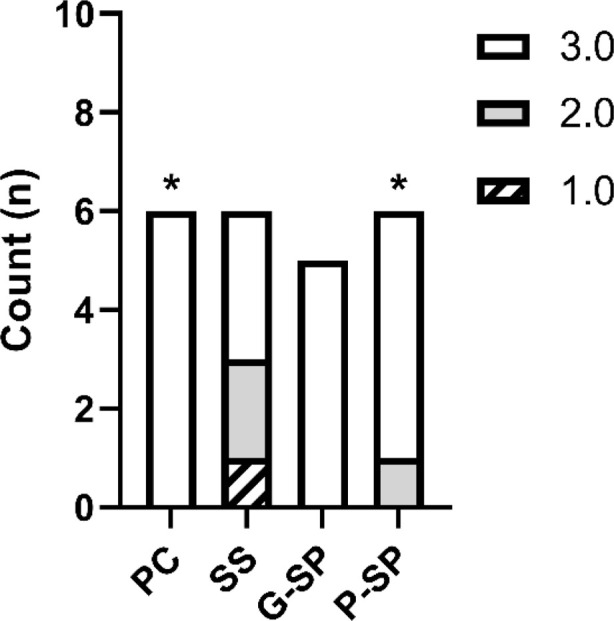
The graph presents the analysis of epithelialization in the affected region among the Positive Control, Saline Solution, SP Gel, and SP Macerate groups. Legend: Epithelialization levels across experimental groups. The re-epithelialization process was assessed using an ordinal scale: Score 1 represents complete epithelialization, Score 2 represents partial epithelialization, and Score 3 corresponds to the absence of epithelialization. A predominance of Score 3 was observed in the Positive Control and Pasta SP (Macerate) groups, while the Gel SP group showed a distribution between Scores 2 and 3. The Saline Solution group exhibited a higher frequency of Score 1, indicating more advanced epithelialization in this treatment. Statistically significant differences were identified between the Saline Solution group and both the Positive Control and Pasta SP groups (p-value = 0.017; p-value <0.05$), demonstrating a superior epithelialization rate for the Saline Solution group under these experimental conditions.

Using the Saline Solution group as the reference, significant differences were also observed versus the Positive Control group (*p* = 0.017) and the SP Macerate group (*p* = 0.022). The comparison with SP Gel (*p* = 0.067) suggested a non-significant trend but did not reach statistical significance (*p* > 0.05). When SP Gel was used as the reference, no pairwise comparison was significant (*p* = 0.531 vs. Positive Control; *p* = 0.067 vs. Saline Solution; *p* = 0.550 vs. SP Macerate). Likewise, when SP Macerate was used as the reference, comparisons with Positive Control (*p* = 1.000) and SP Gel (*p* = 0.550) were not significant, whereas the comparison with Saline Solution was nominally significant (*p* = 0.022), indicating higher epithelialization in SP Macerate relative to Saline Solution before adjustment. As shown in Figure 8, these nominal differences are illustrated in the corresponding graph.

However, after Bonferroni correction for multiple comparisons, none of the contrasts remained statistically significant (all adjusted *p* > 0.05). Therefore, following adjustment for multiple testing, no statistically significant differences were detected among groups with respect to epithelialization.

The histological images illustrate the progression of tissue repair across the different treatment groups. In image A, from the Positive Control (PC) group (hematoxylin and eosin, 10×), a well-defined epidermis and an organized dermis are observed. Image B (20×) further demonstrates preservation of the epidermal layers.

In image C, corresponding to the Saline Solution group (10×), overall tissue organization is maintained; however, the cellular response appears lower than that observed in the PC group. Image D (20×) shows inflammatory cell infiltration and early re-epithelialization, evidenced by the presence of a developed stratum corneum, although inflammatory activity remains apparent.

Images E and F correspond to the SP Gel–treated group. In image E (10×), the dermis appears organized, with preserved adnexal structures, including hair follicles and sebaceous glands. Image F (20×) corroborates these findings, showing an epidermis covered by a stratum corneum, consistent with effective regeneration and histological integrity, and suggesting a favorable effect of SP Gel on the healing process.

**FIGURE 9:**
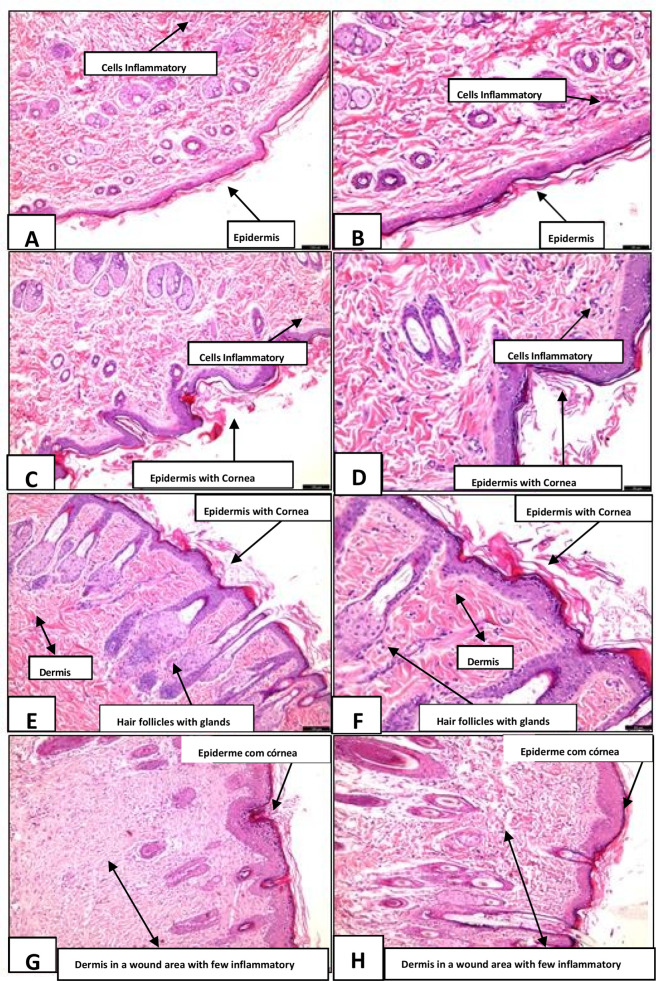
Histological sections stained with hematoxylin and eosin (HE), illustrating tissue repair in the experimental groups. Legend: **(A,B)** (Positive Control–Nebacetin®) show reconstituted epidermis and mild inflammation. **(C,D)** (Saline Solution) display disorganized granulation tissue, intense inflammation, and absence of reepithelialization. **(E,F)** (*S. portulacastrum* gel at 10%) reveal partial epidermis, active fibroblasts, and early collagen organization. **(G,H)** (*S. portulacastrum* macerate) indicate more advanced reepithelialization, higher collagen density, and reduced inflammation, consistent with more mature wound healing.

Finally, images G and H represent the SP Macerate–treated group. In image G (10×), cellular reorganization and possible extracellular matrix deposition are evident, indicating reparative activity. Image H (20×) shows an epidermis with a stratum corneum and mild dermal inflammation, consistent with reduced inflammatory response and progression of tissue repair. Overall, these findings suggest that SP Macerate may contribute to modulation of inflammation and support skin regeneration. As shown in Figure 9.

Histological analysis using Masson’s trichrome staining revealed distinct patterns of tissue regeneration among the experimental groups. In the Positive Control (PC) group, well-distributed blue–green collagen fibers, an intact epidermis, and an organized dermis with preserved skin appendages—including hair follicles and sebaceous glands—were observed. The underlying muscle tissue also appeared preserved, consistent with effective healing. At 20× magnification, densely arranged collagen bundles were evident, indicating a well-established scarring response.

The Saline Solution group exhibited lower collagen deposition, reflected by lighter blue staining. Collagen fibers appeared thinner, with visible fibroblast nuclei, consistent with an earlier reparative stage. The inflammatory response was mild, suggesting limited healing performance under this condition.

The SP Gel–treated group showed relatively homogeneous staining. At 10× magnification, fine collagen fibers were arranged more uniformly, with a modest increase in collagen density. At 20× magnification, collagen fibers appeared organized, and sebaceous glands were preserved, suggesting a potential modulatory effect of SP Gel on extracellular matrix remodeling.

**FIGURE 10:**
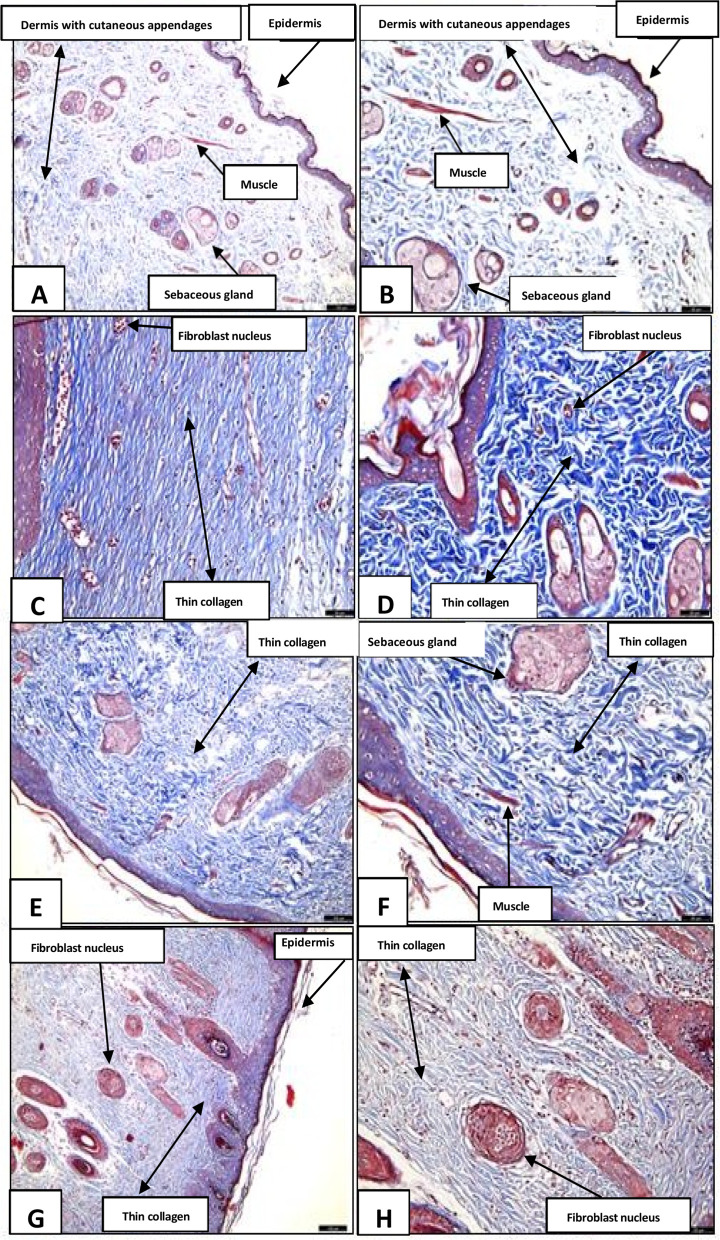
Histological sections stained with Masson’s trichrome highlighting collagen deposition in the treated wounds. Legend: Images **(A,B)** (Positive Control–Nebacetin®) show dense and organized collagen fibers, indicative of mature scar tissue. **(C,D)** (Saline Solution) present sparse and disorganized collagen, suggesting incomplete healing. **(E,F)** (*S. portulacastrum* gel at 10%) reveal reorganizing collagen with moderately distributed fibers. **(G,H)** (*S. portulacastrum* macerate) display intense blue staining and parallel fibers, consistent with an advanced stage of tissue remodeling.

In the SP Macerate–treated group, more intense blue staining in the injured area indicated greater collagen deposition. The epidermis remained well defined, and fibroblast nuclei were evident, suggesting cellular activation. At 20× magnification, fine yet densely organized collagen fibers and clearly distributed fibroblasts were observed, consistent with active tissue regeneration. Overall, these findings suggest that SP Macerate may be associated with a more advanced scarring response than the other treatments, supporting its potential role in extracellular matrix modulation and skin repair. As shown in Figure 10.

## 4 Discussion

Wound healing is a biologically complex process involving coordinated interactions among multiple cell types, chemical mediators, and tissue structures, and it is classically described as comprising four overlapping phases: hemostasis, inflammation, proliferation, and remodeling (Wallace et al., 2023). Medicinal plants have been extensively investigated for wound-healing properties. One review identified 52 species with demonstrated efficacy in experimental models, including *Aloe vera*, *Calendula officinalis*, *Mimosa tenuiflora*, and *Centella asiatica* (Piriz et al., 2014). Other studies have reported anti-inflammatory potential through suppression of inflammatory cytokines in a mixture of *Zingiber officinale*, *Capsicum annuum*, *Curcuma longa*, and *Cinnamomum verum* (Saher et al., 2022; Dev et al., 2019).

The literature has consistently highlighted the phytochemical activity of medicinal plants and, consequently, their potential for diverse therapeutic applications across a wide range of pathological conditions. In this study, we discuss the use of *Sesuvium portulacastrum* leaves and stems in gel and poultice formulations applied to cutaneous wounds, given prior reports describing phytochemicals such as dihydrostigmasterol, epicatechin, and capsaicin, which exert anti-inflammatory and antioxidant effects; gallic and benzoic acids, which show analgesic and anti-inflammatory actions; and alkaloids with cytotoxic and anti-ulcer properties (Al-Azzawi et al., 2012; Mukundh et al., 2024).

Methanolic extracts of the plant have shown *in vitro* antioxidant and anti-inflammatory activity, suggesting mechanisms that may contribute to wound repair by modulating cellular responses involved in tissue regeneration. This evidence supports further investigation of *in vivo* efficacy and potential clinical applications, particularly in resource-limited settings where plant-based therapies may represent accessible and low-cost alternatives (Venkatraman, 2022). However, when translating phytotherapeutic approaches to animal models, it is important to recognise that effectiveness may vary according to factors such as extract preparation, administered dose, lesion type and depth, and host condition (Cury et al., 2013).

The wound healing observed in the present experiment may be associated with phytochemicals such as dihydrostigmasterol, epicatechin, and capsaicin, which exhibit anti-inflammatory and antioxidant effects, as well as gallic and benzoic acids, which have analgesic and anti-inflammatory actions. In addition, these compounds may promote cell proliferation and migration, particularly through capsaicin, consistent with findings reported by Sumioka et al. (2014), who showed that capsaicin doses were capable of promoting corneal wound healing.

These observations underscore the central role of the inflammatory response in tissue repair and indicate that understanding and modulating this initial phase are essential for advancing wound-healing therapies. Accordingly, limiting inflammation as healing progresses—and reducing it in the chronic phase—appears to be important, as proposed by Holzer-Geissler et al. (2022) and corroborated by Xu et al. (2021). These considerations align with cell migration assays conducted using different capsaicin concentrations in skin fibroblasts and cutaneous wounds in a murine model, in which low doses increased cell migration and induced an anti-inflammatory response (Huang et al., 2023). Capsaicin is among the phytochemicals identified in *S. portulacastrum* extracts, accounting for approximately 4.32% of the detected compounds (Al-Azzawi et al., 2012). Nevertheless, caution is warranted when proposing capsaicin-based approaches, as Mukundh et al. (2024) reported a dose-dependent increase in free radical inhibition, whereas Huang et al. (2023) warned that high doses of capsaicin may inhibit cell migration.

Beyond anti-inflammatory activity, the bioactive compounds present in the plant may support cell migration and tissue nutrition through revascularization mediated by epicatechin (Mukundh et al., 2024). This interpretation is consistent with reports indicating stimulation of fibroblast proliferation and increased expression of factors such as TGF-β (transforming growth factor beta) and VEGF (vascular endothelial growth factor), thereby promoting angiogenesis and tissue remodeling (Lyu et al., 2022). These processes are critical for effective wound closure and restoration of skin integrity.

In this study, comparison with Nebacetin®, a topical antibacterial agent, indicated a similar rate and pattern of healing. It should be emphasised, however, that Nebacetin acts primarily as an antimicrobial, an activity that may also be present in *S. portulacastrum* through the combined action of gallic acid, epicatechin, and capsaicin (Mukundh et al., 2024). Thus, the phytotherapeutic formulation may offer a broader profile beyond antimicrobial effects, including anti-inflammatory, antioxidant, and potentially angiogenic actions. In this regard, future comparisons with wound-healing agents with broader mechanisms, such as Fibrase® or *Centella asiatica*–based compounds, may provide additional data relevant to these cellular pathways (Piriz et al., 2014).

Another finding of this study was the high correlation between healing time and reduction in wound size, with correlation coefficients greater than 0.80. This result indicates a strong linear association between the analysed variables, suggesting that wound healing followed a consistent pattern over time. In biomedical research, Pearson correlation coefficient (*r*) values above 0.80 are widely interpreted as indicative of a strong correlation (Field, 2018). Therefore, the data support the reliability of the measurements and indicate that the tested interventions, including those based on *Sesuvium portulacastrum*, were associated with a measurable and well-defined healing trajectory.

Identification of a decreasing linear trend is a favourable indicator, as it suggests continuous and predictable healing without abrupt fluctuations. In tissue regeneration studies, such linear patterns are often associated with controlled physiological processes in which the initial inflammatory response is progressively replaced by new tissue formation and wound re-epithelialization. This finding further supports that all treatments, including those using the plant-based formulations, were compatible with wound repair, even though statistically significant superiority among them was not demonstrated.

Moreover, the high correlation observed suggests that external factors—such as inter-individual variability among rats or environmental influences—had minimal impact on the healing process, indicating that experimental conditions were adequately controlled and that the outcomes likely reflect treatment effects (Montgomery et al., 2021). Rigorous experimental control is essential in animal studies, as stress, diet, environment, and handling can substantially influence healing. Thus, the consistency observed in the data further supports the robustness of the adopted protocol.

Notably, no signs of infection, pain, or behavioural stress were observed in the animals during the study. This finding strengthens the internal validity of the results, as animal welfare is a critical variable in experiments involving live models (Campos et al., 2007). Chronic stress is known to impair healing through the release of glucocorticoids and catecholamines, which can disrupt inflammatory regulation and reduce cell proliferation (Virtanen et al., 2023; Lima et al., 2021). Maintaining a controlled environment and systematically monitoring animal behaviour are therefore essential to ensure the reliability of the findings.

Finally, this study also contributes to the valorization of biodiversity through biotechnology. The rational exploration of native plant species, such as *S. portulacastrum*, can support the development of bio-inputs, pharmaceutical innovation, and sustainable health strategies (Medeiros et al., 2022).

## 5 Conclusion

This study reinforces the feasibility of using plant-derived therapies as sustainable alternatives, highlighting their accessibility, reduced environmental impact, and pharmacological potential. Although no statistically demonstrable superiority was observed, the findings are encouraging and underscore the need for further studies exploring higher-concentration formulations of the plant and/or its combination with other bioactive compounds. The present results may inform the planning and development of new processes, technologies, or therapeutics aimed at improving wound management.

## Data Availability

The original contributions presented in the study are included in the article/supplementary material, further inquiries can be directed to the corresponding author.

## References

[B1] Al-Azzawi A. Alguboori A. Hachim M. Y. Najat M. Al Shaimaa A. Sad M. (2012). Preliminary phytochemical and antibacterial screening of *Sesuvium portulacastrum* in the United Arab Emirates. Pharmacogn. Res. 4 (4), 219–224. 10.4103/0974-8490.102269 23225966 PMC3510875

[B81] Albuquerque U. P. (2010). Implications of ethnobotanical studies on bioprospecting strategies of new drugs in semi-arid regions. Open Complementary Med. J. 2, 21–23. 10.2174/1876391X01002020021

[B2] Barbosa T. A. Gomes F. R. R. (2022). Biodiversidade e conservação da Caatinga: revisão sistemática. J. Environ. Analysis Prog. 7 (4), 177–189. 10.24221/jeap.7.4.2022.5228.177-189

[B3] Campos A. C. L. Borges-Branco A. Groth A. K. (2007). Cicatrização de feridas. ABCD Arq Bras Cir Dig. 20 (1), 51–58. 10.1590/S0102-67202007000100010

[B82] Cordeiro J. M. P. Félix L. P. (2014). Conhecimento botânico medicinal sobre espécies vegetais nativas da caatinga e plantas espontâneas no agreste da Paraíba, Brasil. Rev. Bras. Plantas Med. 16 (3 Suppl. I), 685–692. 10.1590/1983-084X/13_077

[B26] Cury V. Moretti A. I. S. Assis L. Bossini P. Crusca J. D. S. Benatti Neto C. (2013). Low level laser therapy increases angiogenesis in a model of ischemic skin flap in rats mediated by VEGF, HIF-1α and MMP-2. J Photochem Photobiol B 125:164–170. 10.1016/j.jphotobiol.2013.06.004 23831843 PMC3759230

[B6] Dev S. K. Choudhury P. K. Srivastava R. Sharma M. (2019). Antimicrobial, anti-inflammatory and wound healing activity of polyherbal formulation. Biomed. and Pharmacotherapy = Biomedecine and Pharmacotherapie 111, 555–567. 10.1016/j.biopha.2018.12.075 30597309

[B73] Field A. (2018). Discovering statistics using IBM SPSS statistics. 5th edn. Los Angeles/London: SAGE Publications.

[B4] Garcia-Caparros P. Al-Azzawi M. J. Flowers T. J. (2023). Economic uses of salt-tolerant plants. Plants 12, 2669. 10.3390/plants12142669 37514283 PMC10385539

[B95] Grigore M.-N. Vicente O. (2023). Wild halophytes: tools for understanding salt tolerance mechanisms of plants and for adapting agriculture to climate change. Plants 12, 221. 10.3390/plants12020221 36678935 PMC9863273

[B8] Holzer-Geissler J. C. J. Schwingenschuh S. Zacharias M. Einsiedler J. Kainz S. Reisenegger P. (2022). The impact of prolonged inflammation on wound healing. Biomedicines 10 (4), 856. 10.3390/biomedicines10040856 35453606 PMC9025535

[B10] Huang C.-J. Pu C. M. Su S. Y. Lo S. L. Lee C. H. Yen Y. H. (2023). Improvement of wound healing by capsaicin through suppression of the inflammatory response and amelioration of the repair process. Mol. Medicine Reports 28 (2), 155. 10.3892/mmr.2023.13042 37387413 PMC10350740

[B11] Lyu K. Liu X. Jiang X. Chen Y. Lu J. Zhu B. (2022). The functions and mechanisms of low-level laser therapy in tendon repair (review). Front Physiol. 13, 808374. 10.3389/fphys.2022.808374 35242050 PMC8886125

[B12] Lima G. C. Costa M. A. S. Silva Júnior J. B. Silva R. B. Souza I. A. Oliveira A. F. M. (2021). Perfil fitoquímico, atividades citotóxica e genotóxica do extrato aquoso de Rhizophora mangle L. Brazilian Journal of Development. 7 (3), 26458–26474. 10.34117/bjdv7n3-377

[B76] Magwa M. L. Gundidza M. Gweru N. Humphrey G. (2006). Chemical composition and biological activities of essential oil from the leaves of *Sesuvium portulacastrum* . J. Ethnopharmacol. 103 (1), 85–89. 10.1016/j.jep.2005.07.024 16243465

[B32] Meng X. Zhou J. Sui N. (2018). Mechanisms of salt tolerance in halophytes: current understanding and recent advances. Open Life Sci. 13, 149–154. 10.1515/biol-2018-0020 33817080 PMC7874743

[B14] Medeiros J. R. Carvalho A. P. Gomes R. F. (2022). Biotecnologia e valorização da biodiversidade no desenvolvimento de fitoterápicos. Rev. Bras. Biotecnol. 33 (1), 77–89.

[B97] Mehmood A. Javid S. Khan M. F. Ahmad K. S. Mustafa A. (2022). *In vitro* total phenolics, total flavonoids, antioxidant and antibacterial activities of selected medicinal plants using different solvent systems. BMC Chem. 16, 64. 10.1186/s13065-022-00858-2 36030245 PMC9419333

[B15] Montgomery D. C. Runger G. C. Hubele N. F. (2021). Estatística aplicada e probabilidade para engenheiros. 9a ed.

[B16] Mukundh S. T. Veeraraghavan V. P. Panneerselvam S. Jayaraman S. (2024). *Sesuvium portulacastrum* potentiates anticancer activity by facilitating the expression of IRS-1/AKT signalling: an *in vitro* study. J. Pharm. Bioallied Sci. 16 (Suppl. 2), S1270–S1273. 10.4103/jpbs.jpbs_587_23 38882817 PMC11174261

[B17] Nascimento-Júnior B. J. Lima F. M. G. A. Rocha C. R. A. Anunciação C. R. Gonçalves R. K. S. Souto L. B. (2021). Percepções sobre o uso de plantas medicinais por profissionais de áreas rurais e urbanas em cidade no nordeste do Brasil. Rev Fitos 15 (2), 231–241. 10.32712/2446-4775.2021.1048

[B18] Pacheco M. G. N. (2021). Uso de plantas medicinais no tratamento ou como adjuvantes na saúde ginecológica: uma revisão de literatura (Trabalho de Conclusão de Curso). Natal: Universidade Federal do Rio Grande do Norte.

[B78] Purushothaman R. Vishnuram G. Ramanathan T. (2024). Fractionation and identification of bioactive compounds from a salt marsh plant *Sesuvium portulacastrum* (L.) and its antioxidant activity. Nat. Prod. Res. 39, 3455–3458. 10.1080/14786419.2024.2338812 38598319

[B20] Piriz M. A. Lima C. A. B. Jardim V. M. R. Mesquita M. K. Souza A. D. Z. Heck R. M. (2014). Plantas medicinais no processo de cicatrização de feridas: uma revisão de literatura. Rev Bras Pl Med. 16 (3), 628–636.

[B21] Rahman M. M. Mostofa M. G. Keya S. S. Siddiqui M. N. Ansary M. M. U. Das A. K. (2021). Adaptive mechanisms of halophytes and their potential in improving salinity tolerance in plants. Int. J. Mol. Sci. 22 (19), 10733. 10.3390/ijms221910733 34639074 PMC8509322

[B22] Rodrigues F. T. S. de Sousa C. N. S. Ximenes N. C. Almeida A. B. Cabral L. M. Patrocínio C. F. V. (2017). Effects of standard ethanolic extract from *Erythrina velutina* in acute cerebral ischemia in mice. Biomed. and Pharmacother. 96, 1230–1239. 10.1016/j.biopha.2017.11.093 29174035

[B23] Roque A. A. Rocha R. M. Loiola M. I. B. (2010). Uso e diversidade de plantas medicinais da Caatinga na comunidade rural de Laginhas, município de Caicó, Rio Grande do Norte (nordeste do Brasil). Rev Bras Plantas Med. 12 (1), 31–42.

[B24] Saher T. Manzoor R. Abbas K. Mudassir J. Wazir M. A. Ali E. (2022). Analgesic and anti-inflammatory properties of two hydrogel formulations comprising polyherbal extract. J. Pain Research 15, 1203–1219. 10.2147/JPR.S351921 35502403 PMC9056049

[B28] Sumioka T. Okada Y. Reinach P. S. Shirai K. Miyajima M. Yamanaka O. (2014). Impairment of corneal epithelial wound healing in a TRPV1-deficient mouse. Investigative Ophthalmology and Visual Science 55 (5), 3295–3302. 10.1167/iovs.13-13077 24781945

[B99] Valdivieso-Ugarte M. Gomez-Llorente C. Plaza-Díaz J. Gil Á. (2019). Antimicrobial, antioxidant, and immunomodulatory properties of essential oils: a systematic review. Nutrients 11 (11), 2786. 10.3390/nu11112786 31731683 PMC6893664

[B29] Venkatraman A. (2022). Pharmacognosy determination and *in vitro* antioxidant, anti-inflammatory, antimycobacterial activity of salt marsh plants: *sesuvium portulacastrum* and *Salicornia brachiata* . J. King Abdulaziz Univ. Mar. Sci. 32 (1), 1–19. 10.4197/mar.32-1.1

[B195] Virtanen M. I. Brinchmann M. F. Patel D. M. Iversen M. H. (2023). Chronic stress negatively impacts wound healing, welfare, and stress regulation in internally tagged Atlantic salmon *(Salmo salar)* . Front Physiol 14, 1147235. 10.3389/fphys.2023.1147235 37078022 PMC10106625

[B30] Wallace H. A. Basehore B. M. Zito P. M. (2023). “Wound healing phases,” in StatPearls. Treasure island (FL) (Bethesda: StatPearls Publishing). 29262065

[B80] Wu Z. Hu Y. Hao R. Li R. Lu X. Itale M. W. (2025). Research progress of genomics applications in secondary metabolites of medicinal plants: a case study in safflower. Int. J. Mol. Sci. 26, 3867. 10.3390/ijms26083867 40332590 PMC12027854

[B31] Xu Z. Liang B. Tian J. Wu J. (2021). Anti-inflammation biomaterial platforms for chronic wound healing. Biomaterials Science 9 (12), 4388–4409. 10.1039/d1bm00637a 34013915

